# FIG4-Related Parkinsonism and the Particularities of the I41T Mutation: A Review of the Literature

**DOI:** 10.3390/genes15101344

**Published:** 2024-10-21

**Authors:** Iro Boura, Irene Areti Giannopoulou, Vasiliki Pavlaki, Georgia Xiromerisiou, Panayiotis Mitsias, Cleanthe Spanaki

**Affiliations:** 1 School of Medicine, University of Crete, Crete, 70013 Heraklion, Greece; boura.iro@gmail.com (I.B.); renata.giannopoulou@gmail.com (I.A.G.); pavlakivasiliki11@gmail.com (V.P.); p.mitsias@gmail.com (P.M.); 2 Department of Basic and Clinical Neuroscience, Institute of Psychiatry, Psychology & Neuroscience, King’s College London, London SE5 9RT, UK; 3 Department of Neurology, University General Hospital of Heraklion, Crete, 71500 Heraklion, Greece; 4 School of Medicine, University of Thessaly, 41500 Larissa, Greece; georgiaxiromerisiou@gmail.com; 5 Department of Neurology, University General Hospital of Larissa, 41334 Larissa, Greece; 6 Department of Neurology, Henry Ford Hospital, Detroit, MI 48202, USA; 7 School of Medicine, Wayne State University, Detroit, MI 48202, USA

**Keywords:** *FIG4*, parkinsonism, Parkinson’s disease, polyneuropathy, Charcot-Marie-Tooth, genetic

## Abstract

**Background/Objectives**: The genetic underpinnings of Parkinson’s disease (PD) and parkinsonism have drawn increasing attention in recent years. Mutations in the Factor-Induced Gene 4 (*FIG4)* have been implicated in various neurological disorders, including Charcot-Marie-Tooth disease type 4J (CMT4J), amyotrophic lateral sclerosis (ALS), and Yunis-Varón syndrome. This review aims to explore the association between *FIG4* mutations and parkinsonism, with a specific focus on the rare missense mutation p.Ile41Thr (I41T). **Methods**: We identified 12 cases from 10 different families in which parkinsonism was reported in conjunction with CMT4J polyneuropathy. All cases involved the I41T mutation in a compound heterozygous state, combined with a *FIG4* loss-of-function mutation. Data from clinical observations, neuroimaging studies, and genetic analyses were evaluated to understand the characteristics of parkinsonism in these patients. **Results**: In all 12 cases, parkinsonism developed either concurrently or following the onset of CMT4J neuropathy, but was never observed in isolation. Cases of both early- and late-onset parkinsonism were identified, reflecting similarities to genetic forms of parkinsonism with autosomal recessive inheritance. Imaging studies, including Dopamine transporter Single Photon Emission Computed Tomography (DaTscan) and brain magnetic resonance imaging (MRI), revealed abnormalities indicative of neurodegeneration, consistent with findings in other neurodegenerative disorders. **Conclusions**: The co-occurrence of parkinsonism with CMT4J in patients carrying the I41T mutation suggests an expanded spectrum of *FIG4*-related disorders, potentially implicating the same molecular mechanisms seen in other neurodegenerative disorders. Further research into *FIG4*-mediated pathways may offer valuable insights into potential therapeutic targets for disorders of both the central and peripheral nervous systems.

## 1. Introduction

The genetic aspects of Parkinson’s disease (PD) and parkinsonism have been under increasingly intense study in recent years. Several genes have been associated with the development of PD or atypical parkinsonian phenotypes, while numerous genetic loci have been related to specific clinical features of PD, such as early symptom onset or cognitive impairment, fueling discussions about phenotype-genotype correlations [1,2,3]. The identification of these genetic loci has deepened our understanding of the underlying mechanisms of neurodegeneration and pointed toward specific cellular process dysfunctions, such as lysosomal and mitochondrial pathways, which may underlie the death of dopaminergic neurons [4]. Additionally, numerous variants appear to act as shared risk factors across various neurodegenerative disorders, emphasizing the implication of fundamental molecular pathways in the pathophysiology of these conditions [5]. 

Mutations of the Factor-Induced Gene 4 (*FIG4)*, located on chromosome 6q21, have been associated with a broad phenotypic spectrum of neurological manifestations, including: (a) the autosomal recessive form of Charcot-Marie-Tooth type 4J (CMT4J) (OMIM 611228), (b) the autosomal dominant form of amyotrophic lateral sclerosis type 11 (ALS11) (OMIM 612577), (c) the autosomal recessive, neurodevelopmental Yunis-Varón syndrome (OMIM 216340), caused by homozygous loss-of-function *FIG4* mutations, (d) autosomal recessive bilateral temporo-occipital polymicrogyria, caused by a homozygous missense *FIG4* mutation (D783V) (OMIM 612691), and (e) autosomal recessive leukoencephalopathy caused by biallelic deleterious *FIG4* mutations.

In a recent publication, Michaelidou et al. reported a case of biallelic *FIG4* mutations in a patient with atypical parkinsonism and CMT4J from the island of Crete [6]. ‘Closed populations’, such as those found on islands, are valuable for studying the genetics of complex disorders, like neuropathies or parkinsonism, as they can shed light on novel genes or rare variants, promoting further exploration of their role. Here, we present a comprehensive review of reported cases of parkinsonism associated with *FIG4* mutations, further expanding the phenotypic heterogeneity attributed to *FIG4*.

## 2. Materials and Methods

We conducted a descriptive review of papers written in English and indexed in the PubMed/MEDLINE database until August 2024, reporting patients who developed parkinsonism and were carriers of *FIG4* mutations. In the initial search strategy, various combinations of the term “*FIG4*” were used alongside “parkinsonism”, “Parkinson’s disease”, “parkinson”, “extrapyramidal”, “tremor”, “neurodegeneration” or “central nervous system”, either as MeSH terms or as plain text. However, due to the limited number of results obtained at first, the search was broadened to be more inclusive. More specifically, we expanded our approach by conducting a separate search using solely the term “*FIG4*” to capture additional literature not identified with the initial search parameters.

Our selection criteria encompassed original research publications, including case reports, which described at least one patient with parkinsonian features who had a confirmed *FIG4* mutation. We incorporated cases of both typical PD and atypical parkinsonism. For each identified patient, the following information was documented, if available: demographics (sex, age at last assessment), parkinsonism characteristics (onset age, presence of rest tremor, asymmetry of parkinsonism, response to dopaminergic therapy), test results for central nervous system (CNS) involvement [brain magnetic resonance imaging (MRI) or Dopamine transporter Single Photon Emission Computed Tomography (DaTscan)], and any additional neurological deficits, such as cognitive impairment. Special care was taken to include other concomitant *FIG4*-related disorders, if present. Given the observed high frequency of *FIG4*-related neuropathy in the identified cases, particular attention was paid to the characteristics of neuropathy, including the following: type of neuropathy, onset age, and presence of motor and/or sensory deficits, hypo-/hyperreflexia, gait impairment, and skeletal deformities.

We also searched for cases of patients with *FIG4* mutations who presented solely with tremor and had no clear report of parkinsonism or bradykinesia, with the intention of examining them separately; however, no such cases were found. Blood-related relatives of the detected *FIG4*-related parkinsonism cases who also exhibited parkinsonian features but lacked genetic testing were excluded.

Two reviewers worked independently to screen the initial list of generated records, retrieve eligible publications, and collect the required elements. The references of any relevant publications were also screened for eligibility. After completing the selection and data collection process, the two reviewers reached a consensus to finalize which publications/cases and items would be included. An attempt to contact the corresponding author of one publication for clarification on a candidate case was unsuccessful. No automation tools were used in either the selection or data collection process.

## 3. Results

**Figure 1:**
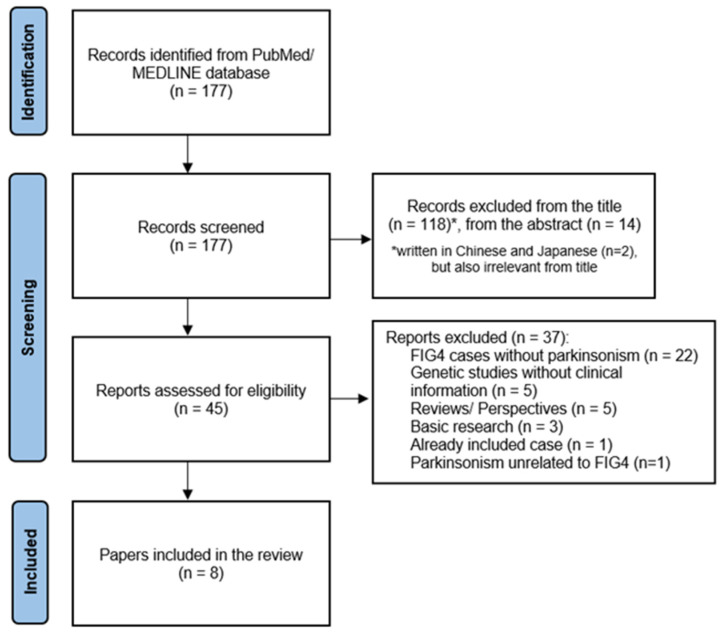
Prisma flowchart for the review of articles on “*FIG4*-related parkinsonism and the particularities of the I41T mutation: a review of the literature” PubMed database on 17 September 2024.

From the search procedure, and after removing duplicates, we screened 177 records. We excluded 118 based on the title and an additional 14 based on the abstract, as they were irrelevant to the scope of our review. We evaluated 45 full-text papers for eligibility and excluded 37 for various reasons (Figure 1).

A total of eight papers were included, describing 12 cases (10 families) that met our criteria [6,7,8,9,10,11,12,13] Reported patients had a mean (±SD) age of 57.4 ± 9.3 years (*n* = 11) at their most recent assessment (Table 1). All patients carried the missense mutation p.Ile41Thr (I41T) in *FIG4* in a compound heterozygous state with a loss-of-function *FIG4* mutation. In all patients, the primary diagnosis was CMT4J polyneuropathy, with parkinsonism developing concurrently or following the onset of neuropathy.

Patients were evenly distributed between those who developed early-onset (<50 years) and those who developed late-onset parkinsonism (between 50 and 66 years of age); two patients had incomplete or missing data. The mean (±SD) onset age of parkinsonism was 50.7 ± 9.8 years. DaTscan was performed in half of the cases, and decreased radiotracer uptake was observed in all instances. Deficits were asymmetric in four patients, reflecting clinical asymmetry of parkinsonian signs in three of them (unavailable data in patient #4), while no asymmetric pattern was detected in patients #5 and #7. Response to dopaminergic therapy was reported for eight patients; five patients responded, and two of them, who were siblings, developed early, peak-dose, troublesome dyskinesia. Two patients exhibited poor response to levodopa, and one patient underwent surgery for deep brain stimulation (DBS) for management of his symptoms; the outcome of this intervention or any information on his past history of dopaminergic medications was not reported. Although the majority of patients with an abnormal DaTscan result exhibited an adequate response to dopaminergic therapy, no consistent pattern was noted between these two parameters. Six CMT4J patients from four nuclear families reported a positive family history of parkinsonism in their siblings, thus suggesting a horizontal pattern of inheritance (autosomal recessive).

Seven patients (64%) developed late-onset CMT4J at a mean (±SD) age of 48 ± 9.1 years, while four patients (36%) presented with early-onset CMT4J, primarily during adolescence. Electrophysiology studies revealed a sensorimotor demyelinating neuropathy in nearly all cases with available data [n = 10 (92%)]. Various skeletal deformities, primarily affecting the feet and toes, were observed in eight patients; no relevant data was available for the remaining cases. The detailed clinical features of the CMT4J polyneuropathy co-existing with parkinsonism in these 12 patients are presented in Table 2.

Cognitive impairment or behavioral problems were detected along with parkinsonism in four patients, with one of them also manifesting epileptic seizures. Findings from brain MRI were abnormal in half of the patients, including localized or diffuse cortical atrophy and pallidal hypointensities, more pronounced on SWI-weighted sequences (Table 1).

## 4. Discussion

Several variants of *FIG4*, which either disrupt or eliminate the function of the *FIG4* protein, have been associated with a wide spectrum of both CNS and peripheral nervous system (PNS) disorders. CMT4J neuropathy and ALS are the most prevalent phenotypes among patients harboring *FIG4* mutations. Adding to the phenotypic heterogeneity of *FIG4*, we review here 12 published cases of CMT4J neuropathy due to *FIG4* mutations that also developed parkinsonian symptoms and signs. Based on a detailed review of their clinical characteristics (where available), we concluded that *FIG4*-related parkinsonism can occasionally be indistinguishable from typical PD, presenting either as a sporadic or a familial extrapyramidal disorder, with a pattern of inheritance suggestive of autosomal recessive transmission.

### 4.1. The I41T Mutation in a Compound Heterozygote State with a Loss-of-Function Mutation Is Consistently Associated with the FIG4-Related Parkinsonism

To date, no patient with subjacent *FIG4* mutations has been reported in the literature as having parkinsonism either in isolation, or in combination with any disorder other than CMT4J. In contrast, all reported cases of patients with *FIG4* mutations who developed parkinsonism also suffered from CMT4J neuropathy. More specifically, they were all compound heterozygotes for the I41T mutation, which is the most frequently encountered variant within the *FIG4* phenotypic spectrum, and various deleterious/loss-of-function mutations in the *FIG4*. No other *FIG4* missense mutation, aside from the I41T, has ever been detected in patients with parkinsonism. Given the emerging significance of the I41T mutation as a genetic variant related to parkinsonian symptomatology, we review below the clinical features of I41T-associated parkinsonian and other phenotypes.

### 4.2. The Heterozygous and Homozygous I41T-Related CMT4J Phenotypes

The presence of biallelic pathogenic *FIG4* variants is the typical underlying genetic cause of the rare demyelinating sensorimotor peripheral neuropathy CMT4J [14] Similarly to the reported patients with *FIG4*-related parkinsonism, CMT4J patients are usually compound heterozygous for a missense and a loss-of-function *FIG4* mutation, with I41T being the most commonly identified missense variant [12,15] Few CMT4J patients were also found to be heterozygous carriers of I41T (18/4000, 0.45%); however, the possibility of overlooked mutations (e.g., deletions) cannot be dismissed [7]

The I41T variant has a population frequency of 0.1% in Europe [7] suggesting that its homozygous state is expected to be quite rare. To date, five homozygous I41T carriers have been reported, all of whom were diagnosed with childhood-onset, demyelinating CMT4J (Table 3) [12,16,17] Despite the early onset of symptoms, half of them exhibited significant clinical stability compared to the compound heterozygous CMT4J cases, whose symptoms usually follow a more aggressive trajectory [16] None of these five patients showed any signs of parkinsonism or other CNS involvement. This observation suggests that the I41T mutation, when present in homozygosity, may be a less aggressive genotype regarding CNS insult compared to compound heterozygous states with a loss-of-function mutation, likely due to differences in *FIG4* protein expression.

### 4.3. The Autosomal Dominant (Heterozygous) I41T-Related ALS11 Phenotype

More recently, numerous heterozygous *FIG4* variants, including two cases carrying the I41T mutation, were identified in patients with familial and sporadic ALS in one American and two large European cohorts, comprising 349, 473, and 201 ALS patients, respectively [18,19,20] The majority of these variants were thought to be causally associated with ALS, either because they were reported as damaging using standard predictive tools, such as SIFT or PolyPhen-2, or due to significantly low minimum allele frequency (MAF < 1%) when compared to the large ethnicity-matched database ExAC (33,370 controls) [19] In all cohorts, most patients exhibited a similar pattern of symptoms, with predominant upper motor neuron signs, slow progression, and longer disease duration, with some of them falling under the sphere of primary lateral sclerosis (PLS), including one patient with the I41T mutation [19] All patients in one of these cohorts exhibited varying levels of brain atrophy, particularly in the frontal region [19] Sensory-motor neuropathy was also confirmed in half of them which, despite being axonal rather than demyelinating, may suggest a genetic link to CMT4J. Two additional cases of I41T heterozygous patients have been reported with an aggressive form of ALS and typical bilateral corticospinal tract hyperintensities in T2/FLAIR sequences, as well as bilateral “motor band sign” on brain MRI [21,22] No ALS patient with either the I41T or alternative *FIG4* mutations has ever been reported to manifest parkinsonian symptoms. The reasons behind this observation remain unclear, further supporting the link of the I41T mutation to parkinsonism, while also suggesting that biallelic deficits may be required for parkinsonian features to emerge.

### 4.4. The I41T-Related Parkinsonian Phenotype

Interestingly, all of the reported parkinsonian patients had a primary diagnosis of CMT4J and exhibited characteristics typical for this condition, including gait impairment and skeletal deformities. Symptoms and signs of neuropathy developed prior to or concurrently with the detection of parkinsonism in all cases, including four early-onset cases presenting during childhood or adolescence. Notably, CMT4J patients with the same *FIG4* genotype, including siblings (patients #2–#3, #4–#5), developed varying levels of neuropathy severity and onset of symptoms, suggesting that additional genetic or environmental factors may influence the expressed phenotype [7]

In contrast to the wide range of onset ages in CMT4J, all reported patients developed parkinsonism during adulthood. The mean age at parkinsonian symptom onset was closer to the lower end of the expected age range for parkinsonism (mainly an age-related disorder), with a considerable number of cases presenting before the age of 50 (n = 5) or even 40 (n = 2). No patient with parkinsonian symptom onset after the age of 66 has been reported so far. This trend of early disease onset is characteristic of genetic cases of parkinsonism. A low threshold for developing peak-dose dyskinesia, both in terms of time and levodopa dose, was observed in two cases belonging to the same family (siblings) (patients #4 and #5), potentially reflecting increased sensitivity to dopaminergic therapy.

In contrast to all reported I41T homozygous CMT4J patients and several compound heterozygous I41T parkinsonian patients who presented with areflexia, patient #1 exhibited asymmetric hyperreflexia. Although this is not uncommon in idiopathic or genetic PD [23,24] it may suggest a shared underlying mechanism of pyramidal tract involvement in both I41T-associated phenotypes of parkinsonism and ALS11. Notably, three cases (patients #1, #7, and #10) presented asymmetric neuropathy findings. Asymmetric progression with disproportionate motor versus sensory PNS involvement has been reported in CMT4J cases [25] Whether this observation indicates a link to the *FIG4*-related ALS spectrum requires further clarification.

DaTscan imaging, where available, was abnormal, suggesting a neurodegenerative parkinsonian disorder. On the other hand, MRI revealed either diffuse or localized brain atrophy in five cases (patients #1, #2, #4–#6). Although abnormal brain MRI findings are relatively uncommon in patients with either CMT4J or PD, these radiological characteristics resemble those reported in *FIG4*-related ALS11 cases [19] Recent literature has further expanded the neuroimaging phenotypic spectrum of *FIG4*, including cases of leukoencephalopathy in patients with biallelic deleterious *FIG4* mutations [26,27] More specifically, three of the *FIG4*-associated leukoencephalopathy cases reported by Lenk et al. were either homozygous or compound heterozygous for the allele c.2459+1G>A (rs747768373) [26] This latter variant was found in patients #4 and #5 (compound heterozygosity with I41T), who also presented with diffuse brain atrophy.

### 4.5. The Pathophysiology of FIG4

The product of *FIG4* is a lipid phosphatase that regulates the homeostasis of phosphatidylinositol 3,5-bisphosphate (PI(3,5)P2) [28] PI(3,5)P2 is a low-abundance phosphoinositide with a crucial role in intracellular signaling, ion homeostasis, and endosomal/lysosomal trafficking, which is considered indispensable for the healthy maturation and maintenance of both the CNS and PNS [29] The *FIG4* phosphatase operates via interaction with the kinase PIKfyve and the scaffold protein VAC14, resulting in the formation of a multi-protein complex at endosomal membranes that regulates the levels of PI(3,5)P2 [30] Hence, a normal *FIG4* protein is a prerequisite for proper endosomal/lysosomal function and trafficking.

The discovery of *FIG4* was made by chance when its inactivation through reverse genetics resulted in the “pale tremor mouse model” [14] These *FIG4*-null mice developed motor symptoms such as tremor, gait difficulties, and abnormal limb postures. Neonatal inactivation of *FIG4* in mouse models caused significant neurodegeneration with vacuolization, hypomyelination in the PNS (which led to the discovery of *FIG4* mutations in patients with CMT4J), and juvenile lethality [14] Lenk et al. demonstrated that the I41T aminoacid substitution resulted in the production of an unstable, but partially functional, *FIG4* phosphatase in vivo due to problematic interaction with VIC14 [15] Increased expression of the defective transcript in transgenic mouse models of the I41T mutation (I41T expression on a *FIG4* null background) could restore *FIG4* protein function and prevent lethality [15]

The above findings support the hypothesis that I41T heterozygosity or homozygosity might be associated with milder forms of CMT4J compared to compound heterozygosity of I41T with a null *FIG4* mutation and biallelic loss-of-function mutations. However, measurements of *FIG4* protein expression in fibroblasts from patients with similar CMT4J severity, who were either homozygous or compound heterozygous for I41T, yielded comparable results [17] Researchers concluded that any mitigating effect of the mutant I41T-*FIG4* protein was negligible. To date, minimal or undetectable levels of *FIG4* protein have been found in western blots from CMT4J patients’ fibroblasts [8,9,31] Whether the phenotypic heterogeneity of CMT4J or other *FIG4*-associated clinical phenotypes is directly linked to *FIG4* protein levels remains unclear.

Although the etiologic connection of *FIG4* mutations to parkinsonism remains to be confirmed, it seems plausible. The abnormal vacuoles detected in the fibroblasts of two related patients with aggressive asymmetric CMT4J harboring the same *FIG4* genotype (I41T and a nonsense mutation) were shown to represent enlarged dysfunctional late endosomes and lysosomes [25] These findings led researchers to suggest impaired endosomal/lysosomal and synaptic function through physical obstruction of intracellular organelle trafficking, pathways that are already implicated in other genetic forms of parkinsonism. Moreover, reduced activity of lysosomal cation channels secondary to reduced PI(3,5)P_2_ was reported to result in osmotic enlargement of the lysosomal compartment and cytoplasmic vacuolization [32] Consequently, *FIG4*-related parkinsonism seems to align with other neurodegeneration- and parkinsonism-related genes, such as *GBA*, which are associated with protein-degrading processes, like the late endosomal/lysosomal pathway. Interestingly, Lewy bodies (mostly brainstem-type, but also cortical) in autopsy-proven cases of PD or dementia with Lewy bodies were found to be immunoreactive for *FIG4*, suggesting that *FIG4* may be involved in the development or breakdown of neuronal inclusions related to neurodegeneration [33] However, no evidence of α-synuclein detection in any biological tissues (e.g., skin, cerebrospinal fluid, blood) was reported for any of the 12 cases of *FIG4*-related parkinsonism.

The important role of *FIG4* protein in PIP_2_ homeostasis and, therefore, in endo-lysosomal function and trafficking aligns well with the molecular pathways known to be disrupted in several genetic forms of PD. A virus-mediated *FIG4*-rescuing therapy that significantly prolongs survival and restores motor performance in animal models of *FIG4* mutations paves the way for the discovery of an effective treatment for this type of genetic parkinsonism [34] As presymptomatic diagnosis via genetic tools is necessary for the timely application of disease-modifying treatments, it is important that clinicians and geneticists become aware of novel genetic types of PD or other parkinsonian syndromes. Genes underlying such parkinsonian phenotypes may reveal new cellular processes or molecular pathways as potential targets for therapeutic interventions [35] In light of these considerations, we suggest that *FIG4* mutations, particularly the I41T mutation, though rare, should be investigated in patients with early-onset parkinsonism and symptoms or signs of unexplained (poly)neuropathy. Additionally, the co-existence of parkinsonism with other CNS and/or PNS neurological phenotypes, either in the same patient or in different members of the same family (in a distribution suggestive of an autosomal recessive mode of inheritance), should raise suspicion of an underlying *FIG4* genetic defect.

Several limitations are acknowledged in our findings. First, the small number of identified patients with *FIG4*-related parkinsonism restricts the ability to draw robust conclusions. While small sample sizes limit the generalizability of any observations, it could be argued that this is an acceptable challenge when studying rare variants, such as the I41T mutation. Moreover, our sample was highly heterogeneous, with significant variability in the reported clinical presentations of parkinsonism, neuropathy, and other manifestations, as well as in the methods used to describe these features. Clinical assessments, including the critical evaluation of response to dopaminergic therapy, and diagnostic tests, such as brain MRI or DaTscan, were not uniformly performed across cases. Additionally, results from standardized assessment tools (e.g., UPDRS) were largely unavailable, hindering direct comparisons between patients. Our efforts to form reliable conclusions were further complicated by the absence of follow-up data and information on disease progression. Despite these limitations, we sought to effectively summarize and group the available information with the aim of proposing potential genotypic and phenotypic patterns, detecting associations with other *FIG4*-related phenotypes, and highlighting gaps in current research that merit further investigation. Our observation that all reported cases of *FIG4*-related parkinsonism were compound heterozygous for the I41T and a loss-of-function *FIG4* mutation highlights such a trend. On the other hand, whether this new, *FIG4*-related genetic form of parkinsonism is a synucleinopathy, like typical sporadic PD, remains an open question, as no reported data on α-synuclein immunohistochemistry or seed amplification assays (SAA) in patients’ tissues or biological fluids have been provided.

## 5. Conclusions

To date, 12 cases of *FIG4*-related parkinsonism have been described in the literature, including both PD and atypical parkinsonism. Notably, all reported cases were compound heterozygous for the I41T variant and a loss-of-function *FIG4* mutation, suggesting a potential link between biallelic deficits of *FIG4*, the I41T mutation, and the development of parkinsonism. CMT4J was present in all reported cases, while no patient exhibited parkinsonian features at an age later than 66. Our findings support the inclusion of *FIG4*, particularly the I41T mutation, in focused genetic screening for cases with potential autosomal recessive inheritance and/or concomitant unexplained neuropathy or skeletal deformities, as well as a positive family history of various neurological diseases.

## Figures and Tables

**Table 1:** Characteristics of *FIG4*-related parkinsonism cases.

Family	Sex, Age	Genetic Substrate	Family History	Parkinsonism Features	Onset	DaTscan Result	Dopaminergic Response	Brain MRI	Other Symptoms
1	#1 M, 54y [7]	c.122T>C (p.I41T)/p.F254SfsX7	NR	PD	54y	NR	Good (LD)	Cerebellum & frontal/parietal atrophy	Slowed mentation
2	#2 F, 54y [8]	c.122T>C (p.I41T)/c.1949-10T>G (intronic)	Brother (Case 3)	Asymmetric parkinsonism (+rest tremor R>L)	52y	NR	NR	Diffuse cortical atrophy, mostly L temporal	Mild cognitive impairment, aphasia
2	#3 M, 48y [8]	c.122T>C (p.I41T)/c.1949-10T>G (intronic)	Sister (Case 2)	Parkinsonism	NR	NR	DBS	NR	Behavioral/speech impairment
3	#4 F, 59y [9]	c.122T>C (p.I41T)/c2459+1G>A (intronic)	Sister (Case 5)	Parkinsonism, early generalized severe dyskinesias	41y	Abnormal (asymmetric)	Good (LD)	Diffuse brain atrophy	Mild optic n. atrophy
3	#5 F, 52y [9]	c.122T>C (p.I41T)/c2459+1G>A (intronic)	Sister (Case 4)	Parkinsonism, early facial dyskinesias	46y	Abnormal (symmetric)	Good (LD)	Diffuse brain atrophy, mostly cerebellum	Mild hearing difficulties
4	#6 F, 67y [9]	c.122T>C (p.I41T)/c.2188dup (p.S730Kfs *3)	NR	Asymmetric parkinsonism, postural instability	65y	Abnormal (asymmetric) **	Poor	Global brain atrophy	Dysarthria, frontal/cognitive signs, severe hearing loss, seizures
5	#7 M, 66y [6]	c.122T>C (p.I41T)/c.1795delC (p.H599fs *24)	Brother (SP, MSA or SPS)	Parkinsonism	Late	Abnormal (symmetric) ***	Poor	Unremarkable	Recurrent trigeminal neuralgia
6	#8 F, 51y [10]	c.122T>C (p.I41T)/c.1447C>T (p.R483 *)	NR	PD (+asymmetric rest tremor)	30–40y	Abnormal (asymmetric) **	Good	Unremarkable	NR
7	#9 M, 41y [11]	c.122T>C (p.I41T)/c.1519dup (p.Y507Lfs *10)	NR	Asymmetric parkinsonism (+rest tremor) *	39y	Abnormal (asymmetric) **	Good	Pallidal hypointensities	NR
8	#10 F, 68y [12]	c.122T>C (p.I41T)/c.1185del (p.A3961Lfs *3)	Sister (PD, neuropathy)	Asymmetric parkinsonism (+tremor)	64y	NR	NR	NR	Neuropathic pain
9	#11 M, 69y [12]	c.122T>C (p.I41T)/c.592C>T (p.Gln198 *)	NR	Parkinsonism	45y	NR	NR	NR	NR
10	#12 NR [13]	c.122T>C (p.I41T)/termination mutation	NR	Akinetic-rigid parkinsonism	NR	NR	NR	NR	-

ALS: amyotrophic lateral sclerosis; DaTscan: dopamine transporter Single Photon Emission Computed Tomography; DBS: deep brain stimulation; F: female; L: left; LD: levodopa; M: male; MSA: multiple system atrophy; n.: nerve; NR: not reported; PD: Parkinson’s disease; R: right; SP: spastic paraparesis; SPS: Stiff-person syndrome; y: years. * baseline UPDRS-III = 19; after 2 years UPDRS-III (ON) = 14 & UPDRS-III (OFF) = 29. ** matching asymmetry in parkinsonian clinical manifestations. *** significant deficits, almost abolished radiotracer uptake

**Table 2:** Characteristics of CMT4J in *FIG4*-related parkinsonism.

ID	CMT4J Onset	Motor Impairment	Gait Impairment	Areflexia	Sensory Deficits	Neuropathy Type	Skeletal Deformities
#1 [7]	Late (54y)	Proximal, asymmetric weakness, LL>UL	Unsteady gait (unassisted)	Brisk, asymmetric reflexes	Proprioception/light touch	Severe combined axonal & demyelinating	Pes cavus, hammer toe
#2 [8]	Early (12y)	Distal weakness (UL & LL)	NR	Yes	Vibration (LL)	Sensorimotor demyelinating	NR
#3 [8]	NR	NR	NR	NR	NR	NR	NR
#4 [9]	Late (41y)	NR	NR	NR	NR	Sensorimotor demyelinating	Small stature, deep bridged nose, clubfeet
#5 [9]	Late (42y)	NR	NR	NR	NR	Sensorimotor demyelinating	Small stature, deep bridged nose, clubfeet
#6 [9]	Late (65y)	LL spasticity	Gait impairment	NR	NR	Severe sensorimotor demyelinating	Small stature, deep bridged nose, microcephaly, brachydactylia, clubfeet, claw toes
#7 [6]	Late (46y)	Distal, asymmetric weakness, LL>UL	Steppage gait (cane)	Yes	Superficial, deep (UL & LL)	Sensorimotor demyelinating	NR
#8 [10]	Early (14y)	Distal weakness	Gait impairment (crutches)	NR	NR	Sensorimotor demyelinating	Scoliosis, pes cavus
#9 [11]	Late (39y)	Distal weakness	No gait impairment	NR	Vibration (LL)	Sensorimotor demyelinating	Hammer toes, pes cavus
#10 [12]	Late (49y)	NR	Gait impairment (crutch)	No	Vibration (UL & LL)	Asymmetric, conduction blocks	Pes cavus
#11 [12]	Early (childhood)	Distal weakness	Gait impairment, regular falls, (walking aid)	Yes	NR	Severe motor demyelinating	NR
#12 [13]	Early (<15y)	Progressive	NR	NR	NR	Sensorimotor demyelinating	Pes cavus

L: left; LL: lower limb; NR: not reported; PD: Parkinson’s disease; R: right; UL: upper limbs; y: years.

**Table 3:** Characteristic of CMT4J in patients with homozygous I41T mutations.

Sex, Age at Diagnosis	Onset	Diagnosis	Mobility/Gait at Diagnosis	Areflexia	Sensory Impairment	Skeletal Deformities	Respiratory Symptoms	Non Motor Symptoms
M, 37y [16]	childhood	CMT4J	Distal weakness, inability to run/jump, preserved ambulation	Yes	No	Pes cavus	Yes (GBS, 15y)	NR
M, 15y [16]	9–10y	CMT4J	Distal weakness, inability to run/jump, preserved ambulation	Yes	Hyperesthesia	Scoliosis, pes cavus	No	NR
M, 44y [16]	13mo	CMT4J	Distal weakness, gait impairment, preserved ambulation	No	Superficial (UL/LL), deep (LL)	Severe kyphoscoliosis (arthrodesis), pes cavus	No	Fatigue Pain (cramps)
M, 19y [12]	childhood	CMT4J	Distal weakness, steppage gait, falls, mobility loss, impaired handwriting	Yes	Proprioceptive ataxia	Severe kyphoscoliosis (arthrodesis candidate), pes cavus	Mild respiratory restrictive s.	Fatigue Pain
M, 10y [17]	9y	CMT4J	Chronic, slowly progressive, asymmetric weakness	NR	No	NR	NR	NR

CMT4J: Charcot-Marie-Tooth type 4J; GBS: Guillain-Barré syndrome; LL: lower limb; M: male; NR: not reported; UL: upper limb; y: years.

## References

[1] Bandres-Ciga S., Diez-Fairen M., Kim J.J., Singleton A.B. (2020). Genetics of Parkinson’s disease: An introspection of its journey towards precision medicine. Neurobiol. Dis..

[2] Kim C.Y., Alcalay R.N. (2017). Genetic Forms of Parkinson’s Disease. Semin. Neurol..

[3] Guadagnolo D., Piane M., Torrisi M.R., Pizzuti A., Petrucci S. (2021). Genotype-Phenotype Correlations in Monogenic Parkinson Disease: A Review on Clinical and Molecular Findings. Front. Neurol..

[4] Nguyen M., Wong Y.C., Ysselstein D., Severino A., Krainc D. (2019). Synaptic, Mitochondrial, and Lysosomal Dysfunction in Parkinson’s Disease. Trends Neurosci..

[5] Wainberg M., Andrews S.J., Tripathy S.J. (2023). Shared genetic risk loci between Alzheimer’s disease and related dementias, Parkinson’s disease, and amyotrophic lateral sclerosis. Alzheimer’s Res. Ther..

[6] Michaelidou K., Tsiverdis I., Erimaki S., Papadimitriou D., Amoiridis G., Papadimitriou A., Mitsias P., Zaganas I. (2020). Whole exome sequencing establishes diagnosis of Charcot–Marie–Tooth 4J, 1C, and X1 subtypes. Mol. Genet. Genom. Med..

[7] Nicholson G., Lenk G.M., Reddel S.W., Grant A.E., Towne C.F., Ferguson C.J., Simpson E., Scheuerle A., Yasick M., Hoffman S. (2011). Distinctive genetic and clinical features of CMT4J: A severe neuropathy caused by mutations in the PI(3,5)P_2_ phosphatase *FIG4*. Brain.

[8] Orengo J.P., Khemani P., Day J.W., Li J., Siskind C.E. (2018). Charcot Marie Tooth disease type 4J with complex central nervous system features. Ann. Clin. Transl. Neurol..

[9] Zimmermann M., Schuster S., Boesch S., Korenke G.C., Mohr J., Reichbauer J., Kernstock C., Kotzot D., Spahlinger V., Schüle-Freyer R. (2020). *FIG4* mutations leading to parkinsonism and a phenotypical continuum between CMT4J and Yunis Varón syndrome. Park. Relat. Disord..

[10] Posada I.J., Domínguez-González C. (2020). CMT4J, parkinsonism and a new *FIG4* mutation. Park. Relat. Disord..

[11] Silva L., Freixo J.P., Brandão A.F., Cardoso M., Damásio J. (2023). Imaging in FIG 4—Related Parkinsonism. Mov. Disord. Clin. Pract..

[12] Beloribi-Djefaflia S., Morales R.J., Fatehi F., Isapof A., Servais L., Leonard-Louis S., Michaud M., Verdure P., Gidaro T., Pouget J. (2023). Clinical and genetic features of patients suffering from CMT4J. J. Neurol..

[13] Machado R.I.L., Souza P.V.S., Farias I.B., Badia B.M.L., Filho J., Lima R.J.V., Pinto W., Oliveira A.S.B. (2023). Clinical and Genetic Aspects of Childhood-Onset Demyelinating Charcot-Marie-Tooth’s Disease in Brazil. J. Pediatr. Genet..

[14] Chow C.Y., Zhang Y., Dowling J.J., Jin N., Adamska M., Shiga K., Szigeti K., Shy M.E., Li J., Zhang X. (2007). Mutation of *FIG4* causes neurodegeneration in the pale tremor mouse and patients with CMT4J. Nature.

[15] Lenk G.M., Ferguson C.J., Chow C.Y., Jin N., Jones J.M., Grant A.E., Zolov S.N., Winters J.J., Giger R.J., Dowling J.J. (2011). Pathogenic mechanism of the *FIG4* mutation responsible for Charcot-Marie-Tooth disease CMT4J. PLoS Genet..

[16] Lafontaine M., Lia A.S., Bourthoumieu S., Beauvais-Dzugan H., Derouault P., Arné-Bes M.C., Sarret C., Laffargue F., Magot A., Sturtz F. (2021). Clinical features of homozygous *FIG4*-p.Ile41Thr Charcot-Marie-Tooth 4J patients. Ann. Clin. Transl. Neurol..

[17] Hu B., McCollum M., Ravi V., Arpag S., Moiseev D., Castoro R., Mobley B., Burnette B., Siskind C., Day J. (2018). Myelin abnormality in Charcot-Marie-Tooth type 4J recapitulates features of acquired demyelination. Ann. Neurol..

[18] Chow C.Y., Landers J.E., Bergren S.K., Sapp P.C., Grant A.E., Jones J.M., Everett L., Lenk G.M., McKenna-Yasek D.M., Weisman L.S. (2009). Deleterious variants of *FIG4*, a phosphoinositide phosphatase, in patients with ALS. Am. J. Hum. Genet..

[19] Osmanovic A., Rangnau I., Kosfeld A., Abdulla S., Janssen C., Auber B., Raab P., Preller M., Petri S., Weber R.G. (2017). *FIG4* variants in central European patients with amyotrophic lateral sclerosis: A whole-exome and targeted sequencing study. Eur. J. Hum. Genet..

[20] Cady J., Allred P., Bali T., Pestronk A., Goate A., Miller T.M., Mitra R.D., Ravits J., Harms M.B., Baloh R.H. (2015). Amyotrophic lateral sclerosis onset is influenced by the burden of rare variants in known amyotrophic lateral sclerosis genes. Ann. Neurol..

[21] Mendes Ferreira V., Caetano A., Santos L., Fernandes M. (2024). *FIG4*-associated disease manifesting as rapidly progressive amyotrophic lateral sclerosis. Neurol. Sci..

[22] Bertolin C., Querin G., Bozzoni V., Martinelli I., De Bortoli M., Rampazzo A., Gellera C., Pegoraro E., Sorarù G. (2018). New *FIG4* gene mutations causing aggressive ALS. Eur. J. Neurol..

[23] Hammerstad J.P., Elliott K., Mak E., Schulzer M., Calne S., Calne D.B. (1994). Tendon jerks in Parkinson’s disease. J. Neural. Transm. Park Dis. Dement Sect..

[24] Lohmann E., Periquet M., Bonifati V., Wood N.W., De Michele G., Bonnet A.M., Fraix V., Broussolle E., Horstink M.W., Vidailhet M. (2003). How much phenotypic variation can be attributed to parkin genotype?. Ann. Neurol..

[25] Zhang X., Chow C.Y., Sahenk Z., Shy M.E., Meisler M.H., Li J. (2008). Mutation of *FIG4* causes a rapidly progressive, asymmetric neuronal degeneration. Brain.

[26] Lenk G.M., Berry I.R., Stutterd C.A., Blyth M., Green L., Vadlamani G., Warren D., Craven I., Fanjul-Fernandez M., Rodriguez-Casero V. (2019). Cerebral hypomyelination associated with biallelic variants of *FIG4*. Hum. Mutat..

[27] Sait H., Shambhavi A., Pandey M., Ravichandran D., Phadke S.R. (2023). T2 olivary nuclei hyperintensities: A characteristic neuroimaging finding in *FIG4*-related leukoencephalopathy. Am. J. Med. Genet. A.

[28] Martyn C., Li J. (2013). *FIG4* deficiency: A newly emerged lysosomal storage disorder?. Prog. Neurobiol..

[29] Mironova Y.A., Lin J.P., Kalinski A.L., Huffman L.D., Lenk G.M., Havton L.A., Meisler M.H., Giger R.J. (2018). Protective role of the lipid phosphatase *FIG4* in the adult nervous system. Hum. Mol. Genet..

[30] Burke J.E., Triscott J., Emerling B.M., Hammond G.R.V. (2023). Beyond PI3Ks: Targeting phosphoinositide kinases in disease. Nat. Rev. Drug Discov..

[31] Gentil B.J., O’Ferrall E., Chalk C., Santana L.F., Durham H.D., Massie R. (2017). A New Mutation in *FIG4* Causes a Severe Form of CMT4J Involving TRPV4 in the Pathogenic Cascade. J. Neuropathol. Exp. Neurol..

[32] Wilson Z.N., Scott A.L., Dowell R.D., Odorizzi G. (2018). PI(3,5)Pcontrols vacuole potassium transport to support cellular osmoregulation. Mol. Biol. Cell.

[33] Kon T., Mori F., Tanji K., Miki Y., Toyoshima Y., Yoshida M., Sasaki H., Kakita A., Takahashi H., Wakabayashi K. (2014). ALS-associated protein *FIG4* is localized in Pick and Lewy bodies, and also neuronal nuclear inclusions, in polyglutamine and intranuclear inclusion body diseases. Neuropathology.

[34] Presa M., Bailey R.M., Davis C., Murphy T., Cook J., Walls R., Wilpan H., Bogdanik L., Lenk G.M., Burgess R.W. (2021). AAV9-mediated *FIG4* delivery prolongs life span in Charcot-Marie-Tooth disease type 4J mouse model. J. Clin. Investig..

[35] Bonifati V. (2007). Genetics of parkinsonism. Park. Relat. Disord..

